# Supplementary material for: Morphophysiological Characterisation of Guayule (Parthenium argentatum A. Gray) in Response to Increasing NaCl Concentrations: Phytomanagement and Phytodesalinisation in Arid and Semiarid Areas
Source: Plants (Basel). 2024 Jan 27;13(3):378. doi: 10.3390/plants13030378 (PMC10856980; doi:10.3390/plants13030378)
Supplement: Supplementary file 1 [file plants-13-00378-s001.zip › plants-2806234-supplementary.pdf]

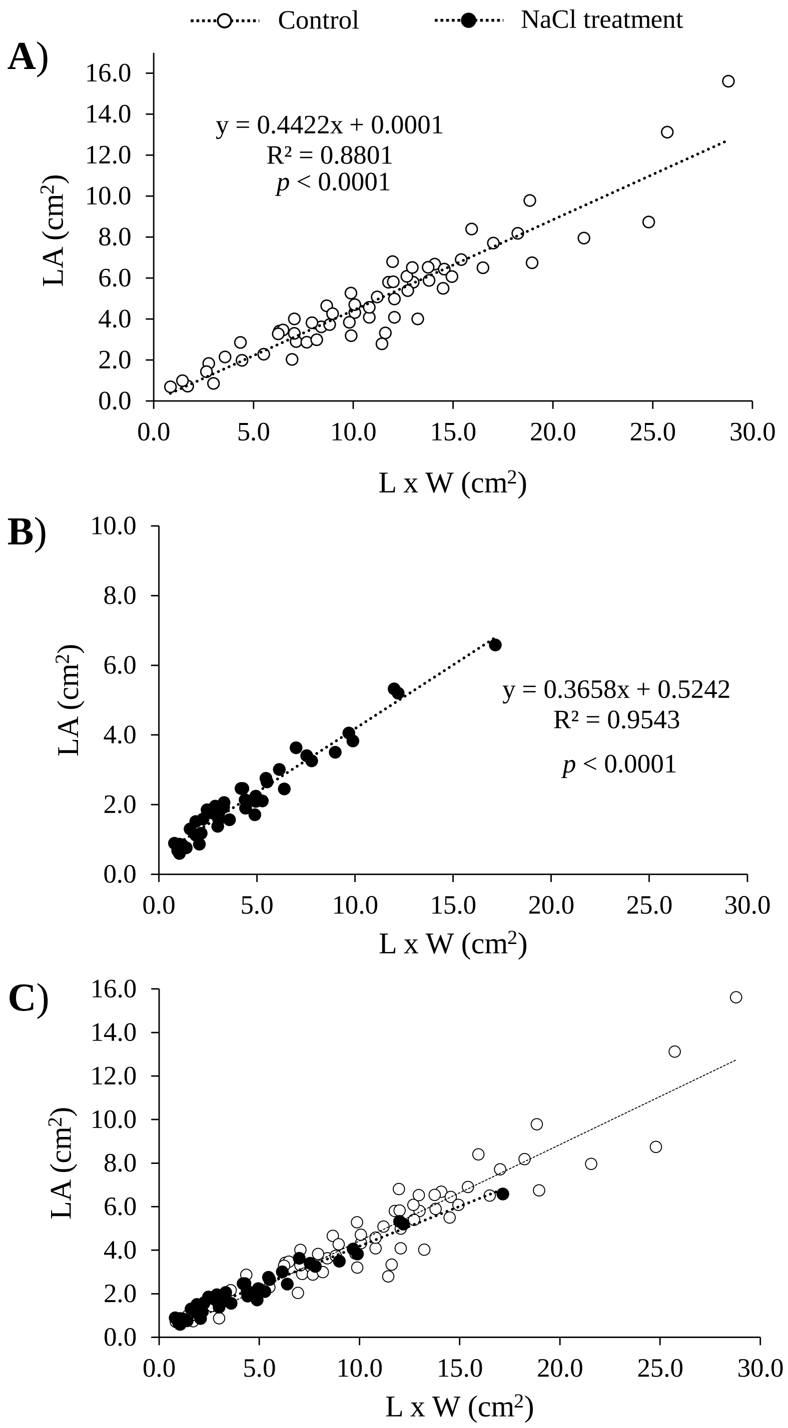

Figure S1. Linear regression models between leaf area (LA, cm^2) and the product of maximum leaf length and width (L x W, cm^2) measured in guayule (*Parthenium argentatum* A. Gray) for **A** – control plants and **B** – plants exposed to 40 g L^{-1} NaCl during the 30 days-hydroponic test. The analysis of covariance (ANCOVA, $p \leq 0.05$) was applied to verify the influence of NaCl treatment on such relation. **C** - Both the slope (p = 0.646) and intercept (p = 0.240) of the two regression were not significantly different between control and NaCl-treated plants. For each regression, the equation, coefficient of determination (R^2) and significance level (p) are shown.

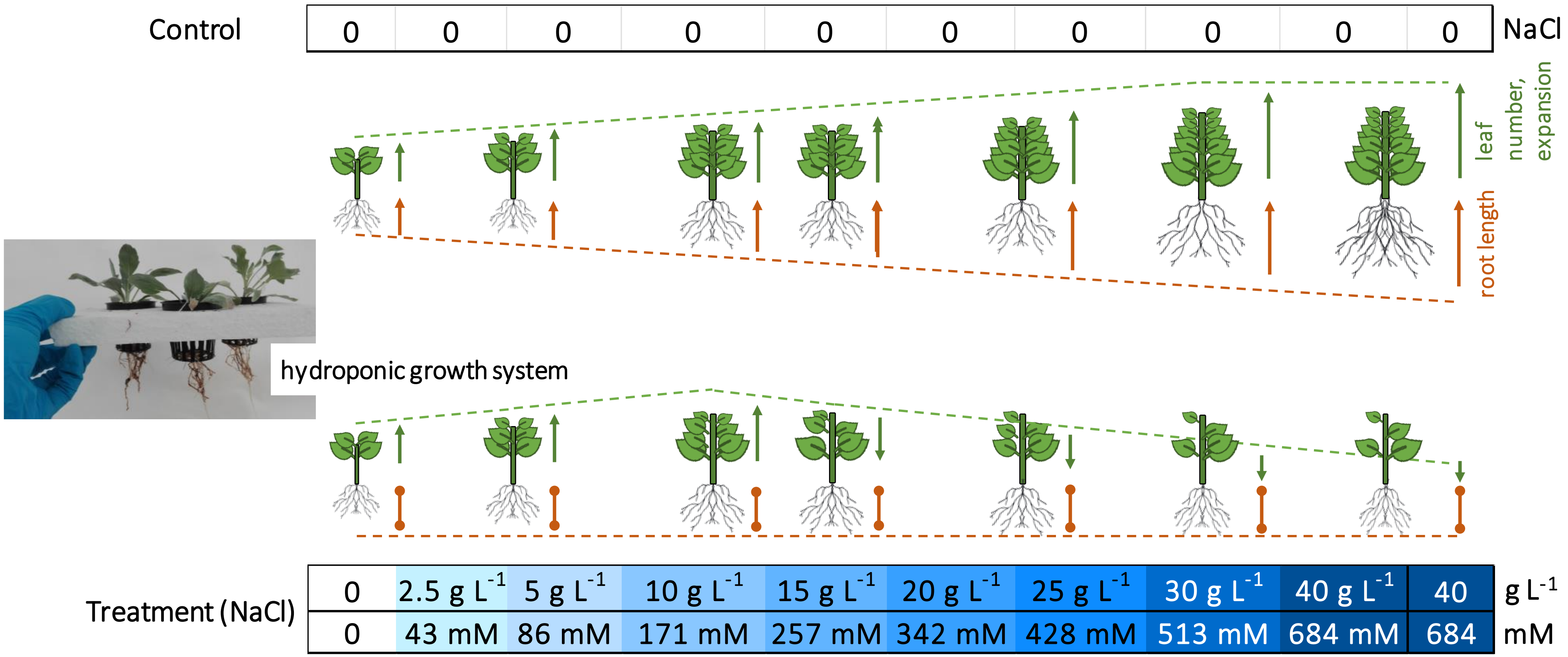

Figure S2. Visual picture illustrating the differences in growth (root length, leaf number and expansion) between control and NaCl-treated plants of guayule exposed to increasing NaCl concentrations (from 2.5 to 40 g L^{-1}, that is from 42.8 to 684.4 mM) in a hydroponic floating root system.

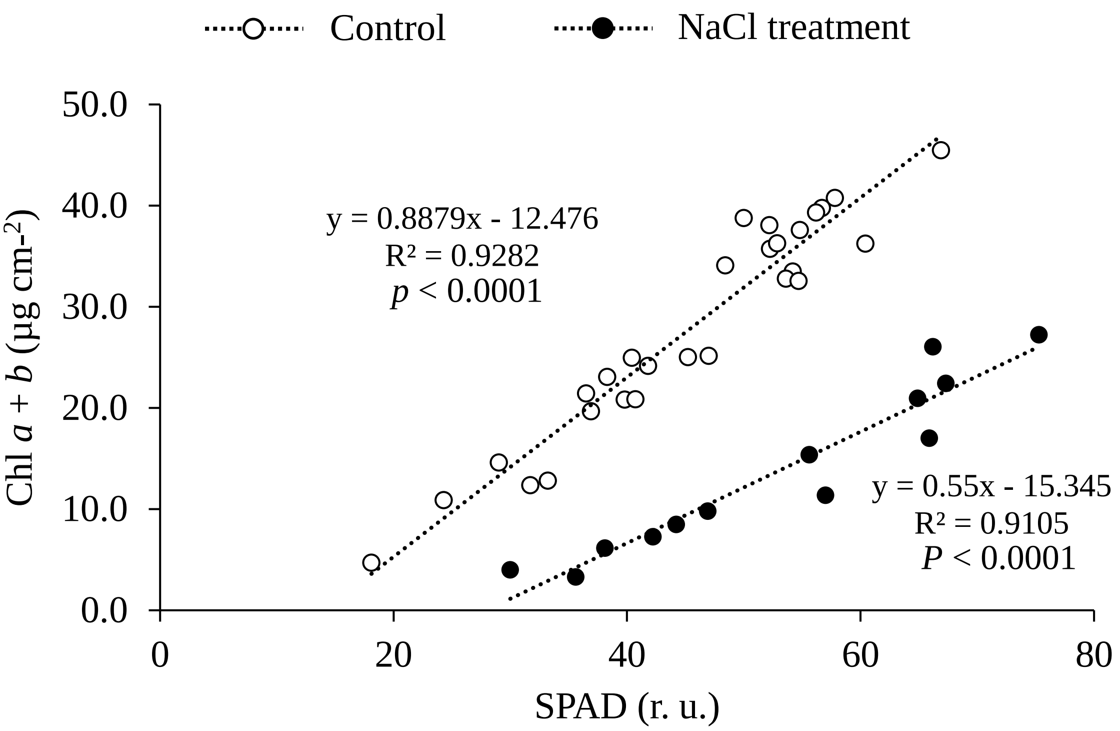

Figure S3. Linear regression models between leaf SPAD-values and total chlorophyll concentration (Chl *a*+*b*) expressed in µg cm^{-2} in guayule control and treated plants exposed to increasing NaCl treatments during the 30 days-hydroponic test. The analysis of covariance (ANCOVA, $p \leq 0.05$) was applied to verify the influence of NaCl treatment on such relations. In the linear regression of controls (white circles) and NaCl treated plants (black circles) the slopes were significantly different (p = 0.012) while the intercepts were not (p = 0.095). The equation, coefficient of determination (R^2) and significance level (p) are shown for each regression; r. u., relative units.

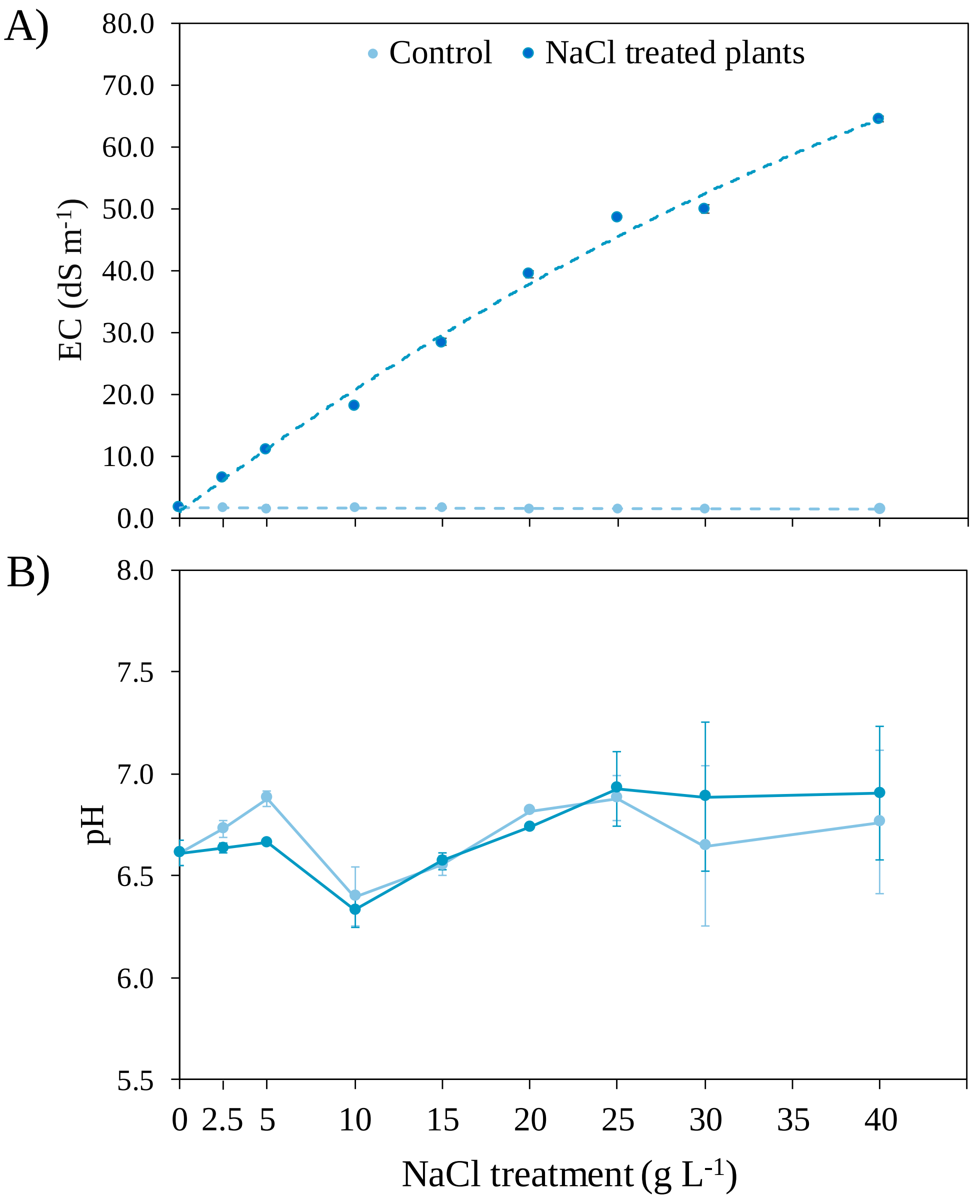

Figure S4. A - Electrical conductivity (EC, dS m^{-1}) and pH (- **B**) monitored in nutrient solutions of controls and treated plants of guayule (*Parthenium argentatum* A. Gray) during the screening test in the hydroponic floating root system. Guayule (about 3.5 months old) was exposed to increasing NaCl concentrations (from 2.5 to 40 g L^{-1}, that is from 42.8 to 684.4 mM) for 30 days according to the scheme of experimental set-up shown in Figure 1). Values are means ± SE of at least three EC and pH determinations performed for each NaCl concentrations tested during the experimental time-course.
